# Integrated metabolomic, proteomic, and transcriptomic analyses reveal the production of bioactive metabolites and antidiabetic effects in mature *Solanum lasiocarpum* fruit

**DOI:** 10.3389/fpls.2026.1774981

**Published:** 2026-03-06

**Authors:** Fangbo Li, Zixiao Jiang, Arunrat Chaveerach, Frédéric Anderson Konkobo, Mohd Kafeel Ahmad Ansari, Gwendolyn Felocity Ban, Mamadou Abdoulaye Konare, Lamin Manjang, Zachary Rochelin, Yang Yang, Nurul Aduka Syameera, Runglawan Sudmoon, Yangyang Liu, Shiou Yih Lee

**Affiliations:** 1 Faculty of Health and Life Sciences, INTI International University, Nilai, Negeri Sembilan, Malaysia; 2 Hainan Provincial Key Laboratory of Resources Conservation and Development of Southern Medicine; International Joint Research Center for Quality of Traditional Chinese Medicine; Institute of Medicinal Plant Development, Chinese Academy of Medical Sciences; Peking Union Medical College, Haikou, China; 3 Faculty of Science, Khon Kaen University, Khon Kaen, Thailand; 4 Laboratory of Biochemistry, Biotechnology, Food Technology and Nutrition (LABIOTAN), Department of Biochemistry, and Microbiology, University Joseph Ki-Zerbo, Ouagadougou, Burkina Faso; 5 Department of Biology, Faculty of Natural Science, University of Guyana, Georgetown, Guyana; 6 School of Agriculture, Papua New Guinea University of Technology, Lae, Morobe, Papua New Guinea; 7 Department of Biology, Faculty of Sciences and Techniques (FST), University of Sciences, Techniques and Technologies of Bamako (USTTB), Bamako, Mali; 8 Catholic Relief Services, Serekunda, Gambia; 9 College of Agriculture and Environmental Science, American University of the Caribbean, Les Cayes, Haiti; 10 Faculty of Liberal Arts, Shinawatra University, Pathum Thani, Thailand; 11 Center of Health, Well-Being, and Environmental Sustainability, INTI International University, Nilai, Negeri Sembilan, Malaysia; 12 Faculty of Law, Khon Kaen University, Khon Kaen, Thailand

**Keywords:** diabetes mellitus, functional foods, human health, shikimate pathway, Terung Dayak

## Abstract

Diabetes mellitus is a chronic metabolic disorder that affects millions of people globally. Among three types of diabetes, Type 2 diabetes mellitus (T2DM) is a rapidly growing global health challenge. Despite available modern antidiabetic drugs, patients still struggle with side effects and treatment failure, as an alternative to this, there is a crucial requirement to develop a potential and traditional plant-based medicine which could be a safer sources and multi-target therapies to treat chronic disease like diabetes. *Solanum lasiocarpum* (*S. lasiocarpum*) is a sour fruit-vegetable being widely used in Southeast Asia as both food and traditional medicine, including for the management of diabetes. However, its active components and antidiabetic mechanisms have not been systematically explored. In this study, we combined metabolomics, proteomics, and transcriptomics to investigate the bioactive pathways and potential molecular targets of *S. lasiocarpum*. Untargeted UHPLC–QTOF–MS profiling identified 45 candidate bioactive compounds with good predicted gastrointestinal absorption, and the network pharmacology analysis linked these compounds to 43 diabetes-related human targets. Protein–protein interaction analysis highlighted several core nodes, including *TNF*, *PPARG*, *IL6*, *AKT1*, and *STAT3*, and functional enrichment suggested roles in hormone regulation, inflammation, glucose and lipid metabolism, and vascular function. *De novo* transcriptome assembly and data-independent acquisition-based proteomics of mature *S. lasiocarpum* fruit showed that central carbon metabolism is highly active and that the shikimate, phenylpropanoid, and flavonoid pathways are strongly expressed at both gene and protein levels. Key enzymes such as *EPSPS*, *PAL*, *C4H*, *4CL*, *CHS*, *CHI*, *F3H*, and *FLS* formed a coherent biosynthetic network supporting sustained production of phenolic and flavonoid metabolites. Integrating these omics layers with target prediction suggests that *S. lasiocarpum* may exert antidiabetic effects by modulating a *TNF–PPARG* axis, reducing pro-inflammatory signaling while supporting insulin-sensitizing pathways. Overall, these results support the traditional use of *S. lasiocarpum* and provide a multi-omics resource to prioritise candidate metabolites, enzymes and targets for follow-up studies. As the pathway links were inferred computationally, the proposed *TNF–PPARG*-centred mechanism should be regarded as hypothesis-generating and will require validation in experimental models and, ultimately, well-designed human intervention trials.

## 1 Introduction

Diabetes mellitus (DM) is one of the most increasing chronic metabolic diseases at an alarming rate worldwide, with an estimated 589 million adults affected in 2024 and projections rising to 853 million by 2050 (Sun et al., 2022). Type 2 diabetes mellitus (T2DM) accounts for over 90% of all cases and is closely linked to lifestyle factors including poor diet, physical inactivity, and population aging (Zheng et al., 2018). Although existing therapies—such as metformin, sulfonylureas, and insulin—remain central to treatment, many patients experience side effects, psychological treatment barriers, or secondary drug failure, often resulting in complex medication regimens (Davies et al., 2022; Bitsang et al., 2025). These challenges highlight the continuing need for safer and multi-target therapeutic options that can widely support long-term metabolic health (Inzucchi et al., 2015).

Medicinal plants have long served as an important resource in global healthcare sector, with approximately 80% of the world’s population depending on herbal remedies (Ekor, 2014; Ansari et al., 2024). Modern analytical tools have provided renewed scientific support for many traditional practices, demonstrating that plant-derived metabolites often act synergistically across multiple biological pathways (Yuan et al., 2016). This multi-target pharmacology is particularly valuable in managing complex diseases like T2DM. Within this context, *Solanum* of Solanaceae family stands out for its diverse biologically active derived substances called phytochemicals. Several members, including *Solanum melongena* and *Solanum nigrum*, have demonstrated antidiabetic effects through antioxidant activity, enzyme inhibition, and glycemic regulation (Das and Barua, 2013; Elekofehinti et al., 2013), encouraging exploration of under-researched species with similar potential.

Among these lesser-studied species, *S. lasiocarpum* Dunal (Terung Asam or Terung Dayak in Malay) is especially noteworthy. It is native to Borneo and widely cultivated across Southeast and South Asia, the fruit holds cultural, culinary, and economic significance in Sarawak, where it is recognized under Geographical Indication (GI2010-00002) for its regional identity and quality attributes (Rushdy et al., 2025). Ethnobotanical records often describe its traditional use for managing fever, inflammation, metabolic conditions, and sore throat, while indigenous communities value it as a nutritious wild vegetable often grown without pesticides, contributing to sustainable food systems (Hoe and Siong, 1999). A number of previous scientific studies have confirmed that the mature fruit of *S. lasiocarpum* is rich in alkaloids, flavonoids, steroids, saponins, terpenoids, tannins and other phenolic compounds with antioxidant, antibacterial, and anticancer properties (Depasthika et al., 2025). More recent work highlights its high chlorogenic acid content, potent antioxidant activity, and notable anti-obesity potential, suggesting its relevance for metabolic disease management beyond traditional use (Saini et al., 2025).

Despite its long history of use and promising phytochemical profile, the molecular mechanisms underlying the biological activities of *S. lasiocarpum* remain poorly understood. Research on this species is still fragmented across diverse sources, and genomic resources are lacking (Ting and Ding, 2021). For non-model plants such as this, integrated multi-omics approaches provide an effective means to uncover active biosynthetic pathways and generate mechanistic insights (Cardona et al., 2024). Recent reviews have summarised how omics strategies can accelerate the discovery of biosynthetic genes and regulatory networks that underpin plant natural product production, especially when genomics, transcriptomics, and metabolite profiling are interpreted together. These reviews also highlight emerging approaches such as single cell sequencing, spatial transcriptomics, mass spectrometry imaging, and machine learning, which are expected to further improve pathway discovery and functional annotation in non model plant species (Wan et al., 2025). Building on ethnomedicinal knowledge and preliminary *in vitro* evidence, this study presents the first comprehensive multi-omics analysis of mature *S. lasiocarpum* fruit. By combining metabolomics, transcriptomics, and proteomics, the aim of this study was to establish a foundational molecular database, identify pathways relevant to its antidiabetic potential, and provide a platform for future targeted research, compound isolation, and functional validation. Alongside pharmacotherapy, dietary strategies and functional foods are increasingly explored as complementary approaches for long-term metabolic control. Recent evidence and synthesis work has emphasised that whole-food matrices and their fermentation products can deliver combinations of phytochemicals that act across oxidative stress, inflammation, insulin sensitivity and the gut microbiome, thereby offering multi-pathway effects that are difficult to capture with single-compound interventions (Nair et al., 2025). In line with this view, a clinical and computational investigation of red date fruit vinegar reported synergistic links between fermentation-derived constituents and interconnected cardiovascular and T2DM-related pathways, highlighting the value of systems-level analyses when evaluating functional foods (Ali et al., 2025). From a methodological standpoint, foodomics and multi-omics integration provide a practical route to connect phytochemical diversity with molecular mechanisms, allowing studies to move from descriptive profiling towards testable hypotheses (Mahato et al., 2024). Importantly, pairing metabolomics with physiological phenotyping can help bridge prediction and validation, as shown by targeted metabolomics studies in diabetic mice where phenylpropanoid-rich plant materials were mechanistically linked to metabolic improvement via gut microbiota modulation (Cheng et al., 2025).

## 2 Materials and methods

### 2.1 Plant material collection and preparation

Fresh *S. lasiocarpum* fruits were harvested in March 2025, from three mature plants grown in the Herbal garden of INTI International University, Malaysia. The fruits were at their fully mature stage, characterized by a vibrant yellow-orange skin and firm pulp. Upon harvesting, the tiny hairs present on its skin surface were washed off thoroughly with deionized water. Fruits were then dried using C-fold towels. For the fruit samples used in RNA extraction, the fruit was cut into smaller pieces to fit in a 50 mL centrifuge tube containing NucleoProtect RNA (Macherey-Nagel, Germany) to preserve the specimen prior to total RNA extraction. All specimens were prepared in three biological replicates.

### 2.2 Untargeted metabolomics

#### 2.2.1 Sample preparation

Approximately 100 mg of fruit tissue was pulverized with the help of liquid nitrogen using mortar and pestle. The sample powder was mixed with 1 mL of pre-chilled extraction solution (methanol: acetonitrile: water = 2:2:1, v/v/v) and vortexed thoroughly. The mixture was then subjected to ultrasonic treatment in an ice-water bath for 10 min, followed by rapid freezing in liquid nitrogen for 1 min. This freeze-thaw cycle was repeated three times to enhance metabolite extraction. The sample was incubated at −20 °C for 1 h, then centrifuged at 14, 000 g at 4°C for 15 min. The supernatant was collected and dried under nitrogen. The dried residue was reconstituted in 100 μL of acetonitrile: water (1:1, v/v), vortexed for 30 s, and sonicated in an ice-water bath for 10 min. After a final centrifugation at 14, 000 g at 4°C for 15 min, the supernatant was transferred to LC-MS vials for analysis.

#### 2.2.2 UHPLC-QTOF-MS analysis

Chromatographic separation was performed using a Waters ACQUITY UPLC system coupled to an AB Sciex TripleTOF 5600+ quadrupole time-of-flight mass spectrometer. An ACQUITY UPLC BEH Amide column (100 mm × 2.1 mm, 1.7 μm particle size) was used for hydrophilic interaction chromatography (HILIC), with the column temperature maintained at 25°C. Mobile phase A consisted of 25 mM ammonium hydroxide (NH_4_OH) and 25 mM ammonium acetate (NH_4_OAc) in water, and mobile phase B was acetonitrile. The gradient program (at 0.30 mL/min flow rate) was as follows: 0–1 min, 95% B; 1–14 min, linear decrease from 95% to 65% B; 14–16 min, linear decrease from 65% to 40% B; 16–18 min, hold at 40% B; 18–18.1 min, return to 95% B; 18.1–23 min, equilibration at 95% B. The injection volume was 2 μL, and the sample sequence was randomized.

The electrospray ionization (ESI) source parameters were set as follows: Gas1 (nebulizer gas) = 60 psi, Gas2 (auxiliary gas) = 60 psi, curtain gas = 30 psi, source temperature = 600°C, and ion spray voltage = ± 5500 V for positive and negative modes. For full-scan MS acquisition, the *m/z* range was 60–1000 Da with an accumulation time of 0.20 s per spectrum. Information-dependent acquisition (IDA) was used for MS/MS scanning (*m/z* 25–1000 Da, 0.05 s per spectrum) with a collision energy of 35 ± 15 eV and a declustering potential of ±60 V. Isotopes within a 4 Da window were excluded, and up to 10 candidate ions were monitored per cycle.

### 2.3 Transcriptome sequencing

#### 2.3.1 RNA extraction and library construction

Approximately 100 mg of fruit pulp was pulverized under liquid nitrogen. Total RNA was extracted using the RNeasy Plant Mini Kit (QIAGEN, Hilden, Germany) with on-column DNase I digestion to remove genomic DNA. RNA quantity and purity were assessed using a NanoDrop 2000 spectrophotometer (Thermo Fisher Scientific, Waltham, MA, USA), and RNA integrity was evaluated using an Agilent 2100 Bioanalyzer (Agilent Technologies, Santa Clara, CA, USA). Only samples with A260/A280 ratios around 2.0 and RNA Integrity Number (RIN) > 8.0 were used for library preparation.

Poly(A)+ mRNA was enriched using oligo(dT) magnetic beads, fragmented, and reverse transcribed into cDNA. Paired-end libraries were prepared using the TruSeq RNA Library Prep Kit (Illumina, San Diego, CA, USA) and sequenced on an Illumina NovaSeq 6000 platform (Illumina, San Diego, CA, USA) to generate 150-bp paired-end reads.

#### 2.3.2 Read processing and *de novo* assembly

Raw reads were processed using fastp v.0.23.0 (Chen et al., 2018) for adapter trimming and quality filtering. Bases with Q-score below 20 were trimmed, and reads shorter than 50 bp after trimming were discarded. This yielded approximately 17.48 Gb of high-quality reads, with over 94.8% of bases having Q30 quality. As there is no reference genome available for *S. lasiocarpum* at that time, we performed *de novo* transcriptome assembly using Trinity v.2.11.0 (Zhao et al., 2025). Clean reads from all three replicates were pooled and assembled using the de Bruijn graph method. The assembly produced 58, 327 non-redundant unigene sequences with an N50 of 1, 852 bp. Assembly completeness was assessed using BUSCO v.5.2.2 (Manni et al., 2021) against the embryophyta_odb10 database, which showed 95.6% completeness (92.1% complete single-copy, 3.5% complete duplicated).

#### 2.3.3 Functional annotation and quantification

Assembled unigenes were annotated by BLASTx searches (*E*-value < 1e-5) against the NCBI non-redundant protein (NR) database (Sayers et al., 2022), Swiss-Prot database (Boutet et al., 2016) and KEGG protein database (Kanehisa et al., 2023). Gene Ontology (GO) terms were assigned using Blast2GO (Götz et al., 2008) based on NR hits, and protein domains were identified using the Pfam database (Mistry et al., 2021). Gene expression levels were quantified by aligning clean reads back to assembled transcripts using RSEM v.1.3.3 (Li and Dewey, 2011). Transcript abundance for each unigene was expressed as fragments per kilobase of transcript per million mapped reads (FPKM).

### 2.4 Proteomic analysis

#### 2.4.1 Protein extraction and digestion

A total of 3 g of fruit tissue was ground in liquid nitrogen using mortar and pestle. Protein extraction was carried out using an SDS-containing lysis buffer under denaturing conditions. Extracts were reduced with 10 mM dithiothreitol (DTT) at 37°C for 1 h and alkylated with 20 mM iodoacetamide (IAA) in the dark for 30 min. Proteins were digested with trypsin at an enzyme-to-protein ratio of 1:50 overnight at 37°C. Resulting peptides were purified by C18 solid-phase extraction and dried by vacuum evaporation prior to LC-MS analysis.

#### 2.4.2 Data-independent acquisition LC-MS/MS analysis

Peptide samples were reconstituted and analyzed on an Easy-nLC 1200 coupled to a Q Exactive HF-X Orbitrap mass spectrometer (Thermo Fisher Scientific, USA) operated in data-independent acquisition (DIA) mode. Peptides were loaded onto a C18 reversed-phase column and separated with a linear acetonitrile gradient (0.1% formic acid) over 16 min. The mass spectrometer acquired MS1 survey scans at 60, 000 resolution (at m/z 200) over m/z 390 to 1010 (AGC target 1e6, maximum injection time 60 ms), followed by sequential MS/MS scans using 75 contiguous isolation windows of approximately 8 m/z spanning m/z 400 to 1000. MS2 scans were collected at 15, 000 resolution (AGC target 1e6, injection time 20 ms) with higher-energy collisional dissociation (HCD) at normalized collision energy (NCE) 27.

#### 2.4.3 Data processing and protein identification

DIA raw files were processed with DIA-NN v.1.8 (Demichev et al., 2020) in library-free mode against a custom protein database derived from our transcriptome using open reading frames predicted by TransDecoder (Haas et al., 2013). Peptide and protein-level false discovery rates were controlled at 1% using a target-decoy strategy. Protein quantification was based on interference-corrected fragment ion intensities with run-wise normalization. Identified proteins were matched to their corresponding unigenes and annotated with KEGG Orthology (KO) identifiers.

### 2.5 Prediction of active ingredient targets

The SwissTargetPrediction database (https://www.swisstargetprediction.ch/ and ChEMBL database (https://www.ebi.ac.uk/chembl/; (Heinzke et al., 2024) were used to construct a target library for the components of *S. lasiocarpum*. The gene names of the target data were then standardized using the UniProt database (http://www.uniprot.org), and only *Homo sapiens* target data were retained.

### 2.6 Construction of the diabetes-related target set

Disease associated targets for diabetes mellitus were collected from GeneCards (https://www.genecards.org/) and DisGeNET (https://www.disgenet.org/) using the keywords diabetes mellitus and type 2 diabetes. Target names were harmonised to official gene symbols using the UniProt database (https://www.uniprot.org/) to avoid ambiguity across databases. To improve reliability, database-specific relevance filters were applied (e.g., GeneCards relevance score ≥ 15 and DisGeNET score ≥ 0.15), and duplicated entries were removed after merging. The resulting set was used as the diabetes-related target background for downstream intersection and network analyses.

### 2.7 PPI network construction and analysis

To prioritise potential diabetes-relevant targets of *S. lasiocarpum*, the predicted targets of the identified compounds were intersected with the diabetes-related target set described above. The overlapping targets were submitted to the STRING database (https://string-db.org/; Szklarczyk et al., 2023) with *Homo sapiens* selected as the organism. A high-confidence interaction threshold (interaction score ≥ 0.90) was applied to construct the protein–protein interaction (PPI) network, and disconnected nodes were removed to improve interpretability. The PPI network was then imported into Cytoscape v3.10.0 (Shannon et al., 2003) for visualisation and topological analysis. Core targets were prioritised based on node centrality metrics (primarily degree, and where appropriate other measures such as betweenness/closeness), and the highest-ranked nodes were retained for subsequent functional enrichment and pathway interpretation.

## 3 Results

### 3.1 Metabolomics analysis

#### 3.1.1 Chemical composition and confirmation of active ingredients in *S. lasiocarpum*

**Figure 1:**
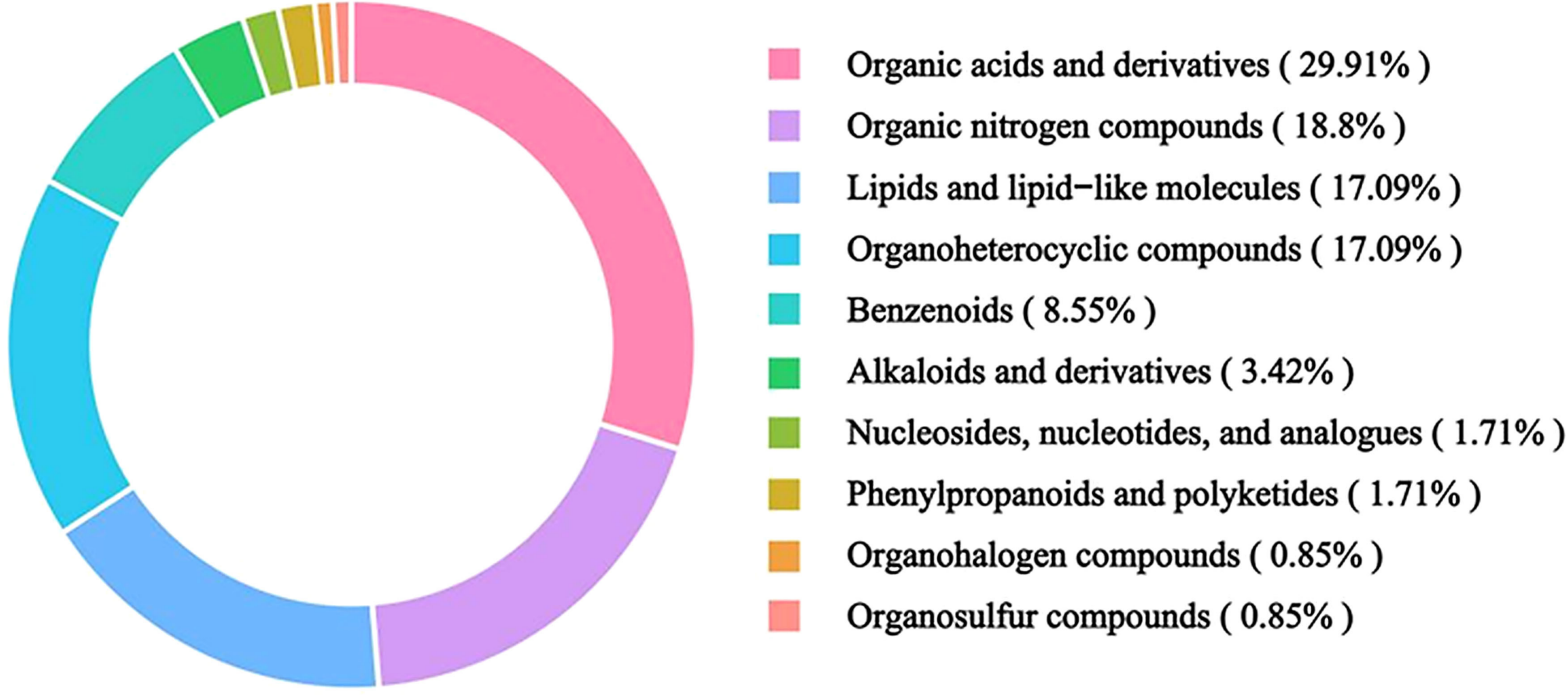
Composition of chemical components of *S. lasiocarpum.*.

Unless otherwise stated, the target–pathway links reported below are derived from in silico prediction and public databases, and should be interpreted as hypothesis-generating rather than direct experimental validation. Untargeted UHPLC–QTOF–MS profiling annotated 45 candidate bioactive compounds that met Lipinski’s rule-of-five and were predicted to have high gastrointestinal absorption (**Supplementary Table S1**). These components include: 13 organic acids and derivatives, nine lipids and lipid-like molecules, seven benzenoids, five organic oxygen compounds, five organoheterocyclic compounds, two organic nitrogen compounds, two nucleosides, nucleotides, and analogues, one alkaloids and derivatives, and one phenylpropanoids and polyketides (**Figure 1**).

Using diabetes as the search term, relevant disease genes were screened based on their compatibility, and genes related to diabetes were integrated from multiple databases. A total of 296 unique gene targets were identified (Relevance score>15). These 45 active components shared a total of 43 unique overlapping targets with diabetes.

#### 3.1.2 PPI network construction and analysis of *S. lasiocarpum*

**Figure 2:**
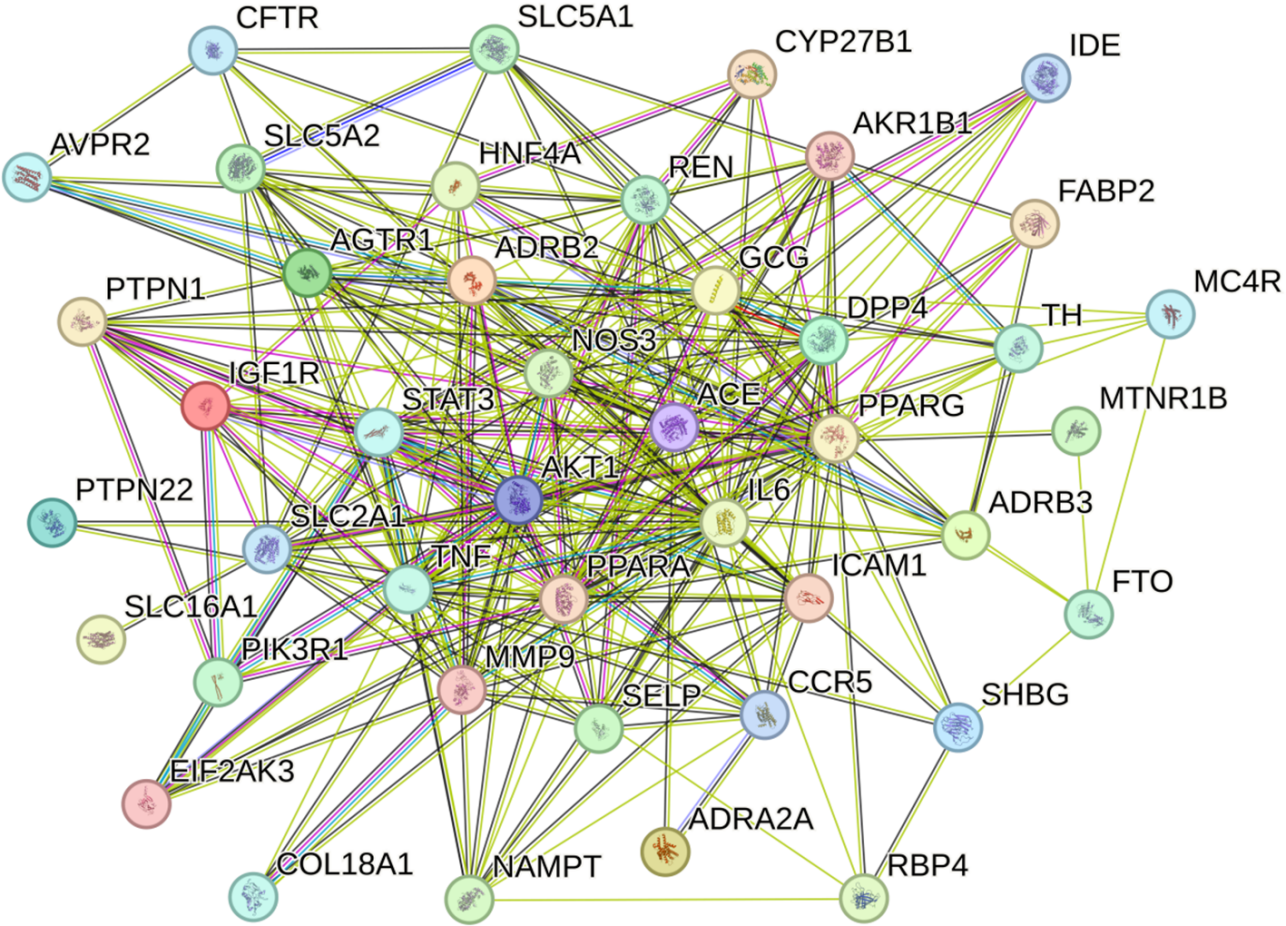
Protein-protein interaction network diagram.

Based on the STRING protein-protein interaction background network, a PPI network was constructed for the 45 active components of *S. lasiocarpum* in the treatment of diabetes, with a confidence threshold of 0.9 to optimize the network construction. In the PPI network of *S. lasiocarpum* in treating diabetes, there were 43 nodes and 276 edges, with an average node degree of 12.8 and an average clustering coefficient of 0.715 (**Figure 2**).

The top 10 core targets with the highest centrality in the treatment of diabetes diseases were identified (**Table 1**). These targets are: *IL6* (34), *PPARG* (32), *TNF* (32), *AKT1* (29), *GCG* (24), *STAT3* (23), *ACE* (22), *MMP9* (22), *REN* (21), and *PPARA* (21).

**Table 1:** Data related to core target screening.

Name	DEGREE	Name	DEGREE
IL6	34	STAT3	23
PPARG	32	ACE	22
TNF	32	MMP9	22
AKT1	29	REN	21
GCG	24	PPARA	21

#### 3.1.3 Gene ontology functional enrichment of the compounds of *S. lasiocarpum*

**Figure 3:**
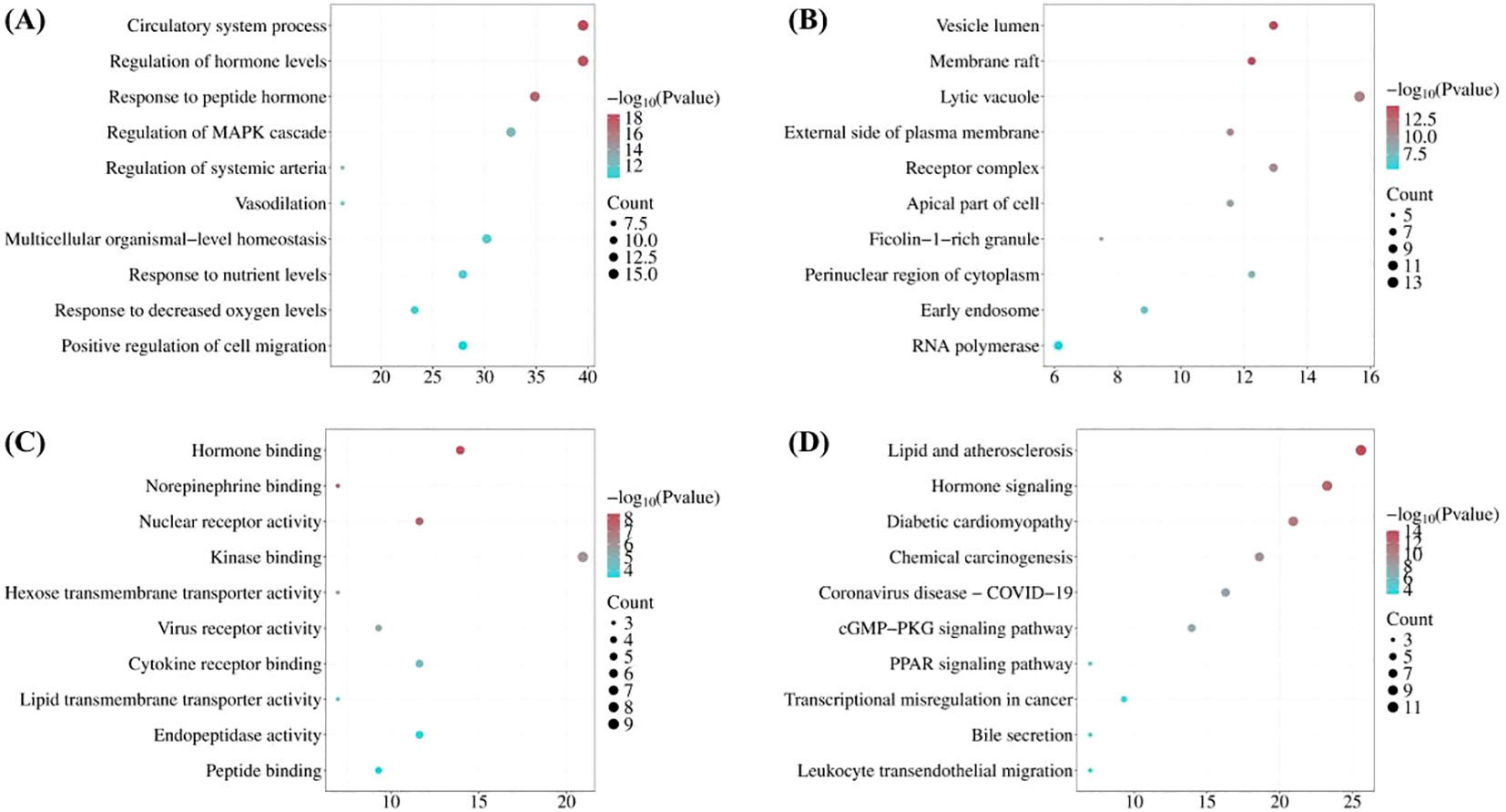
Gene Ontology (GO) functional enrichment and KEGG pathway enrichment analysis **(A)**: GO Biological Processes; **(B)** GO Cellular Components; **(C)** GO Molecular Functions; **(D)** KEGG Pathway).

A potential biological function analysis was conducted for the 45 active component targets of *S. lasiocarpum*. The top 10 items in each category of GO enrichment were visualized based on the *P*-values. In the molecular function analysis, *S. lasiocarpum* was mainly involved in hormone binding (*p* = 4.57 × 10^−09^), norepinephrine binding (*p* = 1.07 × 10^−08^), nuclear receptor activity (*p* = 2.13 × 10^−08^), kinase binding (*p* = 1.20 × 10^−06^), hexose transmembrane transporter activity (*p* = 4.07 × 10^−06^), and others. In terms of biological processes, the active components of *S. lasiocarpum* were primarily involved in circulatory system process (*p* = 3.72 × 10^−19^), regulation of hormone levels (*p* = 8.91 × 10^−19^), response to peptide hormone (*p* = 6.17 × 10^−18^), regulation of MAPK cascade (*p* = 3.72 × 10^−13^), regulation of systemic arterial blood pressure mediated by a chemical signal (*p* = 6.76 × 10^−13^), and more. For cellular components, *S. lasiocarpum* was affecting mainly the vesicle lumen (*p* = 3.89 × 10^−15^), membrane raft (*p* = 5.25 × 10^−15^), lytic vacuole (*p* = 6.46 × 10^−12^), external side of plasma membrane (*p* = 1.02 × 10^−11^), receptor complex (*p* = 2.34 × 10^−11^), and others (**Figure 3**).

### 3.2 Transcriptomic analysis

#### 3.2.1 GO and KEGG enrichment

**Figure 4:**
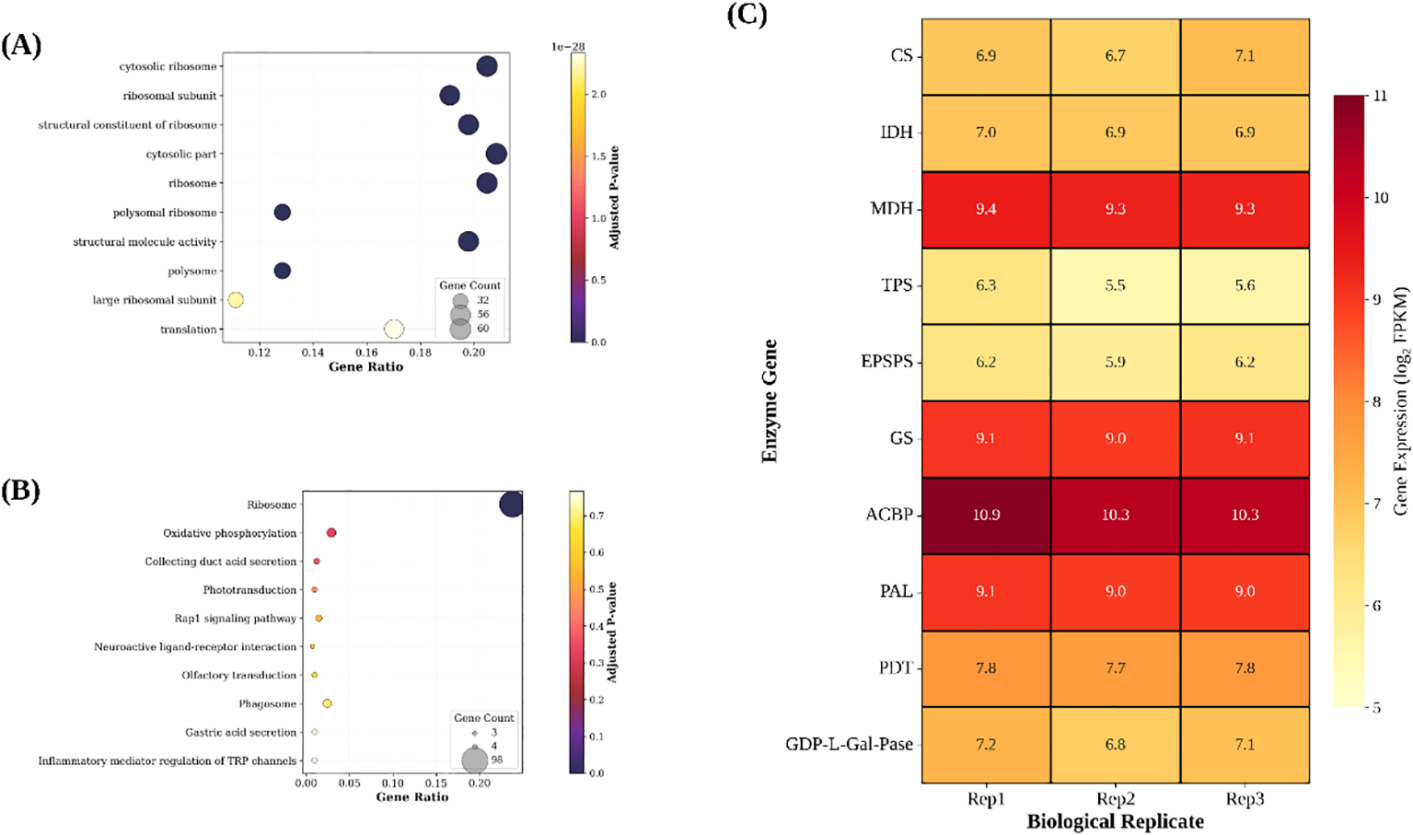
Functional enrichment of the top-500 expressed transcripts. **(A)** GO terms (BP/CC/MF). **(B)** KEGG pathways. **(C)** Expression heatmap of key biosynthetic genes across three biological replicates. Bubble size indicates gene count; color reflects adjusted p-value. Gene expression values are shown as log_2_(FPKM).

To characterize dominant biological processes in mature *S. lasiocarpum* fruit, we performed functional enrichment analysis of the top 500 highly expressed transcripts (mean FPKM: 334 to 12, 010) and present the Top 10 GO terms and Top 10 KEGG pathways (**Figures 4A, B**). GO enrichment was dominated by categories related to protein biosynthesis and energy metabolism. The most significant terms were cytosolic ribosome (adjusted *p* = 1.2 × 10^-14^; 110 genes), ribosomal subunit (adjusted *p* = 3.5 × 10^-14^; 60), structural constituent of ribosome (adjusted *p* = 4.8 × 10^-14^; 52), cytosolic part (adjusted *p* = 6.2 × 10^-14^; 80), and ribosome (adjusted *p* = 7.1 × 10^-14^; 65). Additional enriched terms included translation (adjusted *p* = 1.5×10^-13^; 45), peptide biosynthetic process (adjusted *p* = 2.3 × 10^-13^; 42), structural molecule activity (adjusted *p* = 3.1 × 10^-13^; 48), oxidoreductase activity, and small molecule metabolic process.

KEGG analysis highlighted core carbon and energy metabolism together with precursor-supplying routes. The most significant pathways were Ribosome (adjusted *p* = 1.8 × 10^-14^; 98 genes), Oxidative phosphorylation (adjusted *p* = 4.2 × 10^-3^; 5), Carbon metabolism (adjusted *p* = 8.7 × 10^-3^; 8), Glycolysis/Gluconeogenesis (adjusted *p* = 1.2 x 10^-2^; 6), Amino acid biosynthesis (adjusted *p* = 1.5 × 10^-2^; 7), Starch and sucrose metabolism (adjusted *p* = 2.1 × 10^-2^; 6), Fatty acid biosynthesis and elongation (adjusted *p* = 2.8 × 10^-2^; 4), Ascorbate and aldarate metabolism (adjusted *p* = 3.4 × 10^-2^; 3), and Phenylpropanoid biosynthesis (adjusted *p* = 4.1 × 10^-2^; 5). The enrichment pattern indicates active central metabolism that maintains carbon flux, reducing equivalents, and precursor supply for downstream biosynthesis.

#### 3.2.2 Expression of biosynthetic genes

Annotation of the transcriptome identified key enzymes directly involved both in primary and secondary metabolite biosynthesis. In the tricarboxylic acid (TCA) cycle and organic acid metabolism, citrate synthase (*CS*, g8333_i0) showed mean FPKM of 112.0 across replicates, isocitrate dehydrogenase (*IDH*, g7065_i0) exhibited mean FPKM of 126.3, and malate dehydrogenase (*MDH*, g11827_i0) displayed the highest expression among organic acid enzymes with mean FPKM of 620.4. In carbohydrate metabolism, trehalose-6-phosphate synthase (*TPS*, g1909_i0) was expressed at mean FPKM of 60.3.

For nitrogen assimilation and shikimate pathway genes, glutamine synthetase (*GS*, g4682_i0) demonstrated robust expression with mean FPKM of 552.6. The shikimate pathway bottleneck enzyme 5-enolpyruvylshikimate-3-phosphate synthase (*EPSPS*, g6884_i0) showed mean FPKM of 76.5. At the phenylpropanoid entry point, phenylalanine ammonia-lyase (P*AL*, g4493_i0) and prephenate dehydratase (*PDT*, g8374_i0) exhibited mean FPKM values of 107.1 and 238.0, respectively.

In lipid metabolism, acyl-CoA-binding protein (*ACBP*, g22667_i0) was among the most highly expressed biosynthetic genes with mean FPKM of 1, 490.0. For ascorbate biosynthesis, *GDP-L-galactose* phosphorylase (*GDP-L-Gal-Pase*, g5637_i0) showed mean FPKM of 138.4. These genes displayed consistent expression levels across the three biological replicates, indicating active biosynthetic capacity at the transcriptional level (**Figure 4C**).

#### 3.2.3 Concordance between transcripts and metabolites

To evaluate whether transcript levels of rate-controlling enzymes correspond with metabolite abundance, we selected 10 representative metabolite-gene pairs with well-defined biochemical relationships (**Table 2**). These pairs spanned multiple metabolic pathways including TCA cycle (citrate-*CS*, citrate-*IDH*, L-malic acid-*MDH*), carbohydrate metabolism (trehalose-*TPS*), shikimate pathway (shikimic acid-*EPSPS*), amino acid metabolism (L-glutamine-*GS*), lipid metabolism (palmitic acid-*ACBP*), and phenylpropanoid biosynthesis (benzoic acid-*PAL*, benzoic acid-*PDT*).

**Table 2:** Metabolite–enzyme–gene_ID–FPKM table.

Metabolite	Metabolite_class	Intensity (×10^6^)	Key_enzyme	Full_enzyme_name	Gene_ID	FPKM_mean	Metabolic_pathway
Citrate	Organic acid	4.87	CS	Citrate synthase	g8333_i0	112	TCA cycle
Citrate	Organic acid	4.87	IDH	Isocitrate dehydrogenase	g7065_i0	126	TCA cycle
L-Malic acid	Organic acid	0.513	MDH	Malate dehydrogenase	g11827_i0	620	TCA cycle
Trehalose	Carbohydrate	2.75	TPS	Trehalose-6-phosphate synthase	g1909_i0	60	Trehalose biosynthesis
Shikimic acid	Organic acid	0.352	EPSPS	5-enolpyruvylshikimate-3-phosphate synthase	g6884_i0	77	Shikimate pathway
L-Glutamine	Amino acid	0.0836	GS	Glutamine synthetase	g4682_i0	553	Amino acid metabolism
Palmitic acid	Lipid	0.152	ACBP	Acyl-CoA-binding protein	g22667_i0	1490	Lipid metabolism
Benzoic acid	Aromatic compound	0.0376	PAL	Phenylalanine ammonia-lyase	g14259_i0	476	Phenylpropanoid biosynthesis
Benzoic acid	Aromatic compound	0.0376	PDT	Prephenate dehydratase	g8374_i0	238	Phenylalanine biosynthesis
Galactonic acid	Organic acid	0.0138	GDP-L-Gal-Pase	GDP-L-galactose phosphorylase	g5637_i0	138	Ascorbate biosynthesis

The metabolite-enzyme-gene dataset covered a broad range of expression levels. Key enzymes showed mean FPKM values ranging from 60 (*TPS*, g1909_i0) to 1, 490 (*ACBP*, g22667_i0), while metabolite intensities spanned three orders of magnitude from 0.0138 × 10^5^ (galactonic acid) to 4.87 × 10^5^ (citrate). Among the high-abundance enzyme-metabolite pairs, malate dehydrogenase (*MDH*, g11827_i0, FPKM = 620) corresponded to L-malic acid (intensity = 0.513 × 10^5^), glutamine synthetase (*GS*, g4682_i0, FPKM = 553) corresponded to L-glutamine (intensity = 0.0836 × 10^5^), and acyl-CoA-binding protein (*ACBP*, g22667_i0, FPKM = 1, 490) corresponded to palmitic acid (intensity = 0.152 × 10^5^).

### 3.3 Proteomics analysis

#### 3.3.1 Proteomic results

Data-independent acquisition (DIA) profiling of mature fruit produced a high-confidence proteome. In total, 4, 521 proteins supported by 35, 890 peptides were identified at 1% FDR. The three biological replicates showed strong concordance at the intensity level (mean intensity: Rep1 = 361, 267, Rep2 = 367, 429, Rep3 = 299, 976). To characterize the dominant biochemical functions, enrichment analysis was performed on the most abundant proteins.

**Figure 5:**
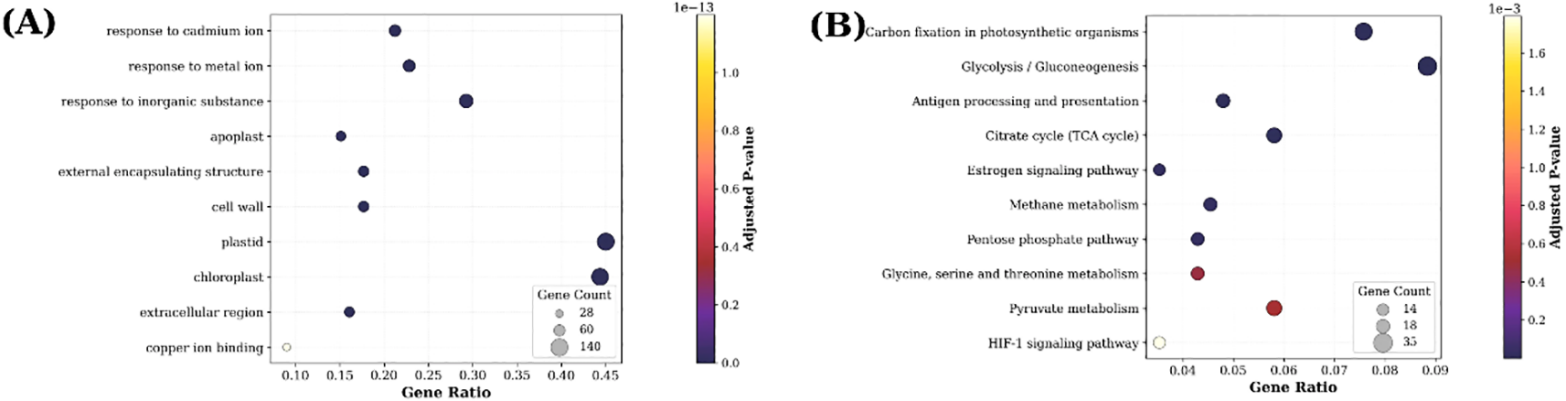
Functional enrichment of abundant proteins from fruit. **(A)** GO top-10 terms showing stress response and structural organization. **(B)** KEGG top-10 pathways highlighting carbon and energy metabolism.

The GO top-10 enrichment profile revealed cellular stress response and structural organization as major themes. Metal ion responses dominated the most significant terms, with response to cadmium ion ranking first (*p* = 2.1 × 10^-29^, 66 genes), followed by response to metal ion (*p* = 2.4 × 10^-26^, 71 genes) and response to inorganic substance (*p* = 1.3 × 10^-25^, 91 genes). Structural and compartment terms were also prominent, including apoplast (*p* = 4.0 × 10^-21^, 47 genes), external encapsulating structure (*p* = 3.3 × 10^-18^, 55 genes), and cell wall (*p* = 4.0 × 10^-18^, 55 genes). Photosynthetic organelles showed high representation with plastid (*p* = 3.2 × 10^-17^, 140 genes) and chloroplast (*p* = 3.5 × 10^-17^, 138 genes). Additional enriched terms included extracellular region (*p* = 8.4 × 10^-16^, 50 genes) and copper ion binding (*p* = 1.2 × 10^-13^, 28 genes) (**Figure 5A**).

The KEGG pathway enrichment revealed strong representation of core carbon and energy metabolism. Carbon fixation in photosynthetic organisms ranked as the most significant pathway (*p* = 5.7 × 10^-12^, 30 genes), followed by Glycolysis/Gluconeogenesis (*p* = 2.4 × 10^-9^, 35 genes) and Citrate cycle (*p* = 2.1 × 10^-8^, 23 genes). These central carbon metabolism pathways collectively involved over 80 proteins. Antigen processing and presentation also showed strong enrichment (*p* = 3.1 × 10^-9^, 19 genes). Additional pathways with notable representation included Estrogen signaling pathway (*p* = 2.3 × 10^-5^, 14 genes), Methane metabolism (*p* = 2.5 × 10^-5^, 18 genes), Pentose phosphate pathway (*p* = 3.1 × 10^-5^, 17 genes), Glycine, serine and threonine metabolism (*p* = 4.8 × 10^-4^, 17 genes), Pyruvate metabolism (*p* = 5.3 × 10^-4^, 23 genes), and HIF-1 signaling pathway (*p* = 1.8 × 10^-3^, 14 genes) (**Figure 5B**). These results indicate that the fruit proteome maintains high metabolic activity and provides precursors for downstream biosynthesis.

#### 3.3.2 Proteomic coverage of key biosynthetic pathways

The identified proteins were mapped onto KEGG reference pathways to assess biosynthetic capacity in *S. lasiocarpum* fruit. The starch and sucrose metabolism pathway (KEGG: ko00500) showed protein detection across multiple enzymatic steps (**Table 3**). Sucrose metabolism involves sucrose-phosphate synthase, sucrose synthase, and invertase. Hexose phosphorylation was represented by hexokinase and glucokinase. Phosphoglucomutase was detected for glucose phosphate interconversion, and UTP-glucose-1-phosphate uridylyltransferase for UDP-glucose formation. Starch biosynthesis included starch synthase and 1, 4-α-glucan branching enzyme. Degradation enzymes comprise -amylase and β-amylase. Trehalose-6-phosphate synthase indicated an additional metabolic branch. The flavonoid biosynthesis pathway (KEGG: ko00941) displayed protein detection from pathway entry through core biosynthetic steps (**Table 4**). Phenylalanine ammonia-lyase initiated the pathway. Downstream enzymes included 4-coumarate-CoA ligase and chalcone synthase for naringenin chalcone formation, with chalcone isomerase enabling conversion to naringenin. Flavanone 3-hydroxylase produced dihydrokaempferol. Branch differentiation involved dihydroflavonol 4-reductase and flavonol synthase. Flavonoid 3’-monooxygenase enabled structural diversification through B-ring hydroxylation. Representative enzymes from both pathways were identified at the protein level, confirming the presence of biosynthetic machinery for these metabolic processes in mature *S. lasiocarpum* fruit.

**Table 3:** Representative enzymes detected in starch and sucrose metabolism (ko00500) with Trinity Gene_IDs and protein-level evidence (DIA identification at 1% FDR).

Enzyme name	Gene symbol	Trinity gene_ID	EC no.	Protein identified	Enzyme name
Hexokinase	HXK	g27810_i1	2.7.1.1	Yes	Hexokinase
Sucrose-phosphate synthase	SPS	g1680_i1	2.4.1.14	Yes	Sucrose-phosphate synthase
Sucrose synthase	SUS	g2888_i1	2.4.1.13	Yes	Sucrose synthase
Invertase (β-fructofuranosidase)	INV	g24053_i0	3.2.1.26	Yes	Invertase (β-fructofuranosidase)
Glucokinase	GK	g2711_i4	2.7.1.2	Yes	Glucokinase
UTP–glucose-1-phosphate uridylyltransferase	UGP	g27721_i0	2.7.7.9	Yes	UTP–glucose-1-phosphate uridylyltransferase
Phosphoglucomutase	PGM	g27721_i1	5.4.2.2	Yes	Phosphoglucomutase
Starch synthase	SS	g10853_i0	2.4.1.21	Yes	Starch synthase
1, 4-α-Glucan branching enzyme	GBE	g24118_i0	2.4.1.18	Yes	1, 4-α-Glucan branching enzyme
Trehalose-6-phosphate synthase	TPS	g24115_i0	2.4.1.15	Yes	Trehalose-6-phosphate synthase
α-Amylase	AMY	g2415_i0	3.2.1.1	Yes	α-Amylase
β-Amylase	BMY	g3211_i0	3.2.1.2	Yes	β-Amylase

**Table 4:** Representative enzymes detected in flavonoid biosynthesis (ko00941) with Trinity Gene_IDs and protein-level evidence (1% FDR).

Enzyme name	Gene symbol	Trinity gene_ID	EC no.	Protein identified	Enzyme name
Phenylalanine ammonia-lyase	PAL	g11109_i0	4.3.1.24	Yes	Phenylalanine ammonia-lyase
4-Coumarate–CoA ligase	4CL	g516_i1	6.2.1.12	Yes	4-Coumarate–CoA ligase
Chalcone synthase	CHS	g25175_i0	2.3.1.74	Yes	Chalcone synthase
Chalcone isomerase	CHI	g5516_i0	5.5.1.6	Yes	Chalcone isomerase
Flavanone 3-hydroxylase (Naringenin 3-dioxygenase)	F3H	g11418_i0	1.14.11.9	Yes	Flavanone 3-hydroxylase (Naringenin 3-dioxygenase)
Dihydroflavonol 4-reductase	DFR	g1145_i0	1.1.1.219	Yes	Dihydroflavonol 4-reductase
Flavonol synthase	FLS	g11420_i0	1.14.20.6	Yes	Flavonol synthase
Flavonoid 3′-monooxygenase	F3′H	g11419_i0	1.14.14.82	Yes	Flavonoid 3′-monooxygenase

### 3.4 Integrated multi-omics analysis

#### 3.4.1 Central carbon metabolism

**Figure 6:**
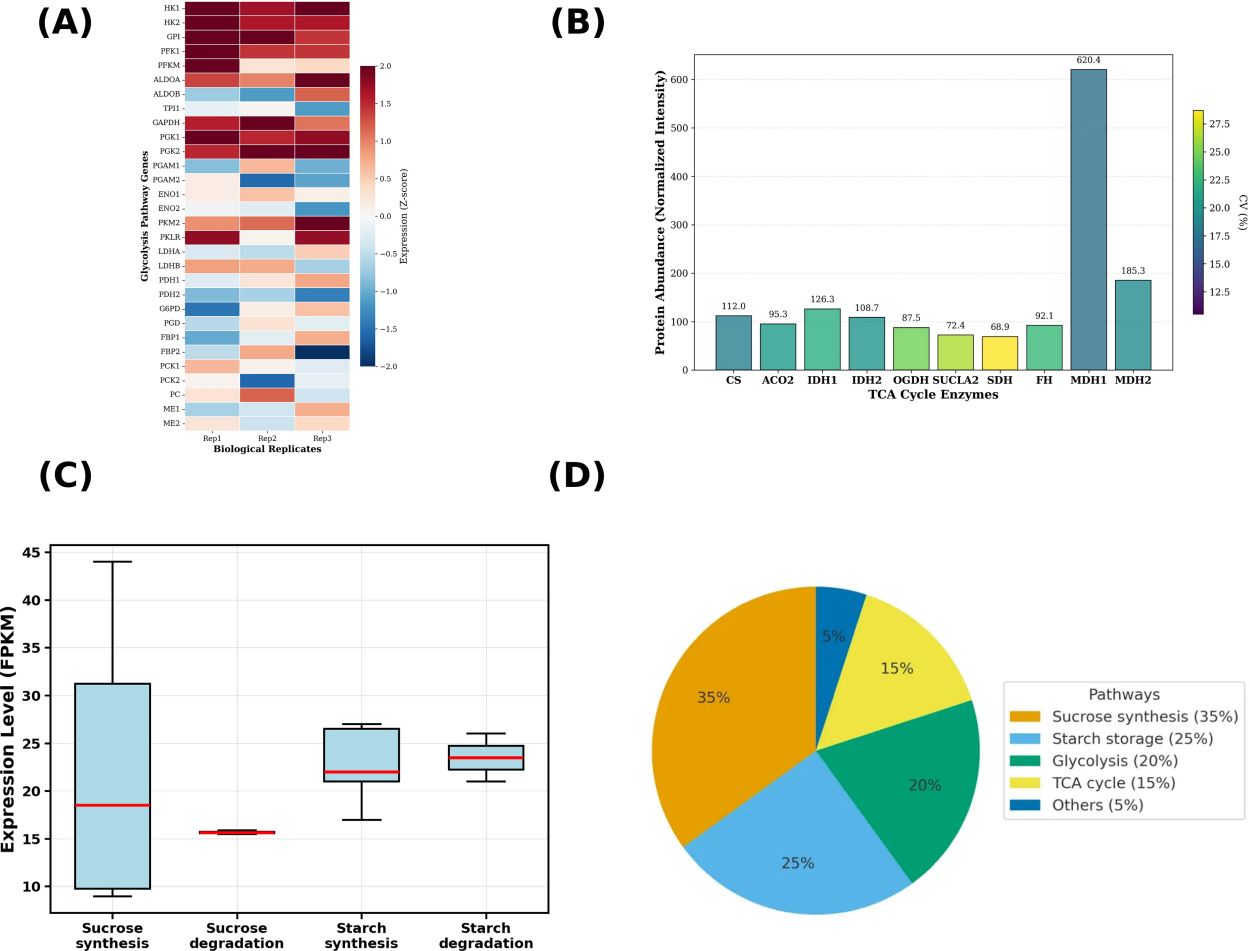
Integrated central carbon metabolism in mature *S. lasiocarpum* fruit. **(A)** Heatmap of glycolysis pathway genes showing Z score normalized transcript abundance across three biological replicates. **(B)** Relative protein abundance of tricarboxylic acid (TCA) cycle enzymes from DIA proteomics, with bars indicating normalized intensity and numbers above bars giving mean values. **(C)** Boxplots of transcript levels for sucrose synthesis, sucrose degradation, starch synthesis, and starch degradation gene sets across three biological replicates. **(D)** Estimated distribution of carbon flux into sucrose synthesis, starch storage, glycolysis, TCA cycle, and other minor routes based on integrated transcriptomic, proteomic, and metabolomic information.

Transcriptome annotation identified 2, 159 genes associated with central carbon metabolism, the majority of which encode key enzymes in glycolysis, the tricarboxylic acid cycle, the pentose phosphate pathway, and starch and sucrose metabolism (**Figure 6A**). In the glycolytic pathway, most rate-limiting enzymes and downstream step genes exhibited highly consistent expression patterns across the three biological replicates. After Z-score standardization, the expression fluctuation of most genes was concentrated within the range of −2 to +2. Key regulatory enzymes such as hexokinase, phosphofructokinase, and pyruvate kinase were generally maintained at medium-high expression levels, suggesting that the glycolytic flux remained relatively active in the fruit. Phosphoglucose isomerase and fructose-bisphosphate aldolase, which catalyze intermediate steps, showed stable and relatively high Z-scores across replicates. Glyceraldehyde-3-phosphate dehydrogenase, enolase and phosphoglycerate kinase displayed highly similar expression patterns, reflecting coordinated transcriptional regulation of multiple reactions in the late glycolytic stage.

Proteomic results provided independent evidence for the activity of mitochondrial respiration in central carbon metabolism (**Figure 6B**). The DIA data detected protein products of all key enzymes in the tricarboxylic acid cycle, including citrate synthase, isocitrate dehydrogenase, α-ketoglutarate dehydrogenase, succinate dehydrogenase, succinyl-CoA synthetase, and malate dehydrogenase, all of which showed clear abundance signals. Protein abundances of citrate synthase and isocitrate dehydrogenase were at medium-high levels among the TCA enzyme group. Malate dehydrogenase exhibited the highest relative abundance, several-fold higher than other enzymes, which may represent an important flux control node in the cycle.

Transcriptional features of sucrose and starch metabolism revealed fine-tuned regulation of carbohydrate partitioning in mature fruit (**Figure 6C**). Genes involved in sucrose synthesis, such as sucrose-phosphate synthase and sucrose-phosphate phosphatase, showed higher expression levels overall than the invertase/sucrose synthase family. In the starch synthesis pathway, starch synthase and starch branching enzyme exhibited similar expression patterns across the three replicates, supporting their synergistic roles in starch chain elongation and branch formation. By contrast, degradative enzymes such as α-amylase and β-amylase generally showed low expression, consistent with the metabolic state of mature fruit being dominated by net starch accumulation. Integrating transcriptomic and proteomic information, we estimated the relative distribution of photosynthetic carbon among different pathways. Approximately 35% of carbon flux entered sucrose synthesis, 25% was stored as starch, 20% fueled glycolysis for energy supply, 15% underwent further oxidation via the TCA cycle, and the remaining 5% was allocated to other metabolic pathways (**Figure 6D**). This carbon flux pattern demonstrates that storage-oriented metabolism centred on sucrose and starch coexists with active respiratory metabolism in mature *S. lasiocarpum* fruit, providing a stable substrate and energy basis for subsequent secondary metabolism.

#### 3.4.2 Lipid metabolism integration

**Figure 7:**
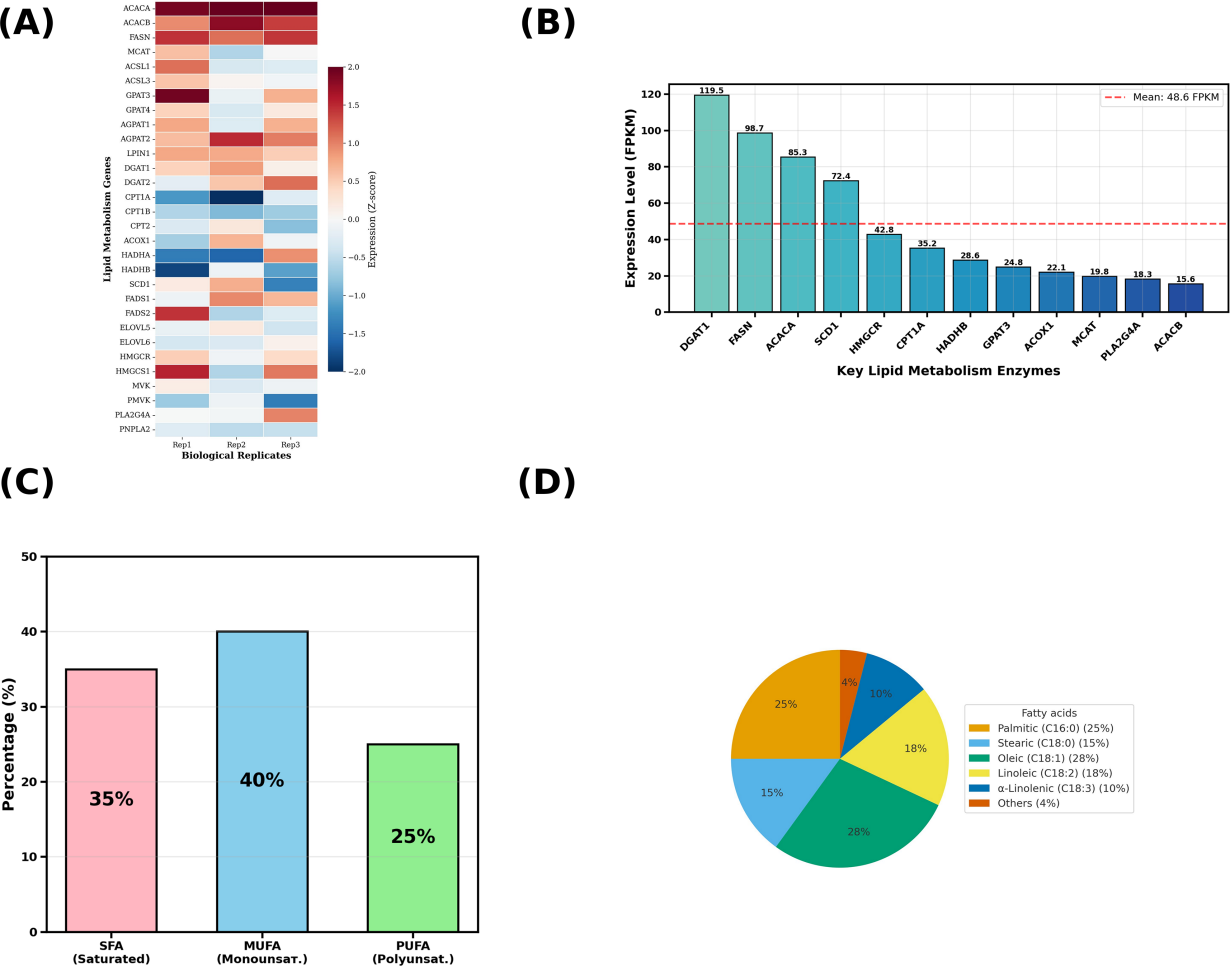
Integrated lipid metabolism in mature *S. lasiocarpum* fruit. **(A)** Heatmap of lipid metabolism genes involved in fatty acid synthesis, elongation, desaturation, and β oxidation, showing Z score normalized transcript abundance across three biological replicates. **(B)** Proportions of saturated fatty acids, monounsaturated fatty acids, and polyunsaturated fatty acids in the total fatty acid pool. **(C)** Composition of major individual fatty acids expressed as percentage contribution to total fatty acids. **(D)** Transcript abundance of key lipid metabolism enzymes, with a red dashed line indicating the mean expression level of this gene set.

At the transcriptomic level, multiple lipid metabolic pathways in *S. lasiocarpum* fruit maintained relatively high activity. Genes related to *de novo* fatty acid synthesis and triacylglycerol synthesis showed consistent expression patterns across the three biological replicates, with Z-scores mainly distributed within the range of −2 to 2 (**Figure 7A**). Acetyl-CoA carboxylase isoforms *ACACA* and *ACACB*, fatty acid synthase *FASN*, and the elongase *ELOVL* family were generally at relatively high expression levels. Genes involved in glycerol backbone acylation, including *GPAT*, *AGPAT* and *LPIN1*, as well as terminal enzymes *DGAT1* and *DGAT2*, also exhibited high expression, suggesting relatively active triacylglycerol synthesis and storage processes. In contrast, genes related to mitochondrial and peroxisomal β-oxidation, such as *CPT1*, *CPT2*, *ACOX1*, *HADHA* and *HADHB*, showed relatively low expression.

Combining FPKM quantification results further delineated the expression hierarchy of key enzymes (**Figure 7B**). Among the selected representative enzymes, *DGAT1* showed the highest average expression, significantly exceeding the average level of the entire lipid metabolism enzyme set. This was followed by *FASN* and *ACACA*, indicating that terminal triacylglycerol synthesis and precursor fatty acid generation represent important flux control nodes in the lipid metabolic network. *SCD1* was at a medium-high expression level and, together with *FADS1* and *FADS2*, supported the synthesis of monounsaturated and polyunsaturated fatty acids. *HMGCR* also exhibited high expression, indicating that the mevalonate pathway and sterol-related metabolism remained quite active in the fruit. Enzymes of β-oxidation such as *CPT1A*, *HADHB*, *ACOX1* and *MCAT* maintained moderate levels, consistent with the metabolic state of moderate degradation and storage-predominant metabolism.

Fatty acid composition analysis was highly consistent with the transcriptome profile. From the perspective of saturation distribution, saturated fatty acids, monounsaturated fatty acids and polyunsaturated fatty acids accounted for approximately 35%, 40% and 25%, respectively. Monounsaturated fatty acids held a slight predominance (**Figure 7C**). Among specific fatty acid species, the two saturated fatty acids C16:0 and C18:0 together accounted for about 40% of total fatty acids. Palmitic acid accounted for approximately 25% and stearic acid for approximately 15%. C18:1 accounted for about 28% and was the most abundant monounsaturated fatty acid. Linoleic acid and α-linolenic acid accounted for approximately 18% and 10%, respectively. Other long-chain and trace fatty acids accounted for about 4% (**Figure 7D**). This fatty acid composition dominated by C18:1, C18:2, and C18:3, is consistent with the medium-high expression of *de novo* synthesis and desaturation-related genes, such as *FASN*, *SCD1*, and *FADS1*/*FADS2*, suggesting that mature fruit possesses both energy storage capacity and potential nutritional value.

**Figure 8:**
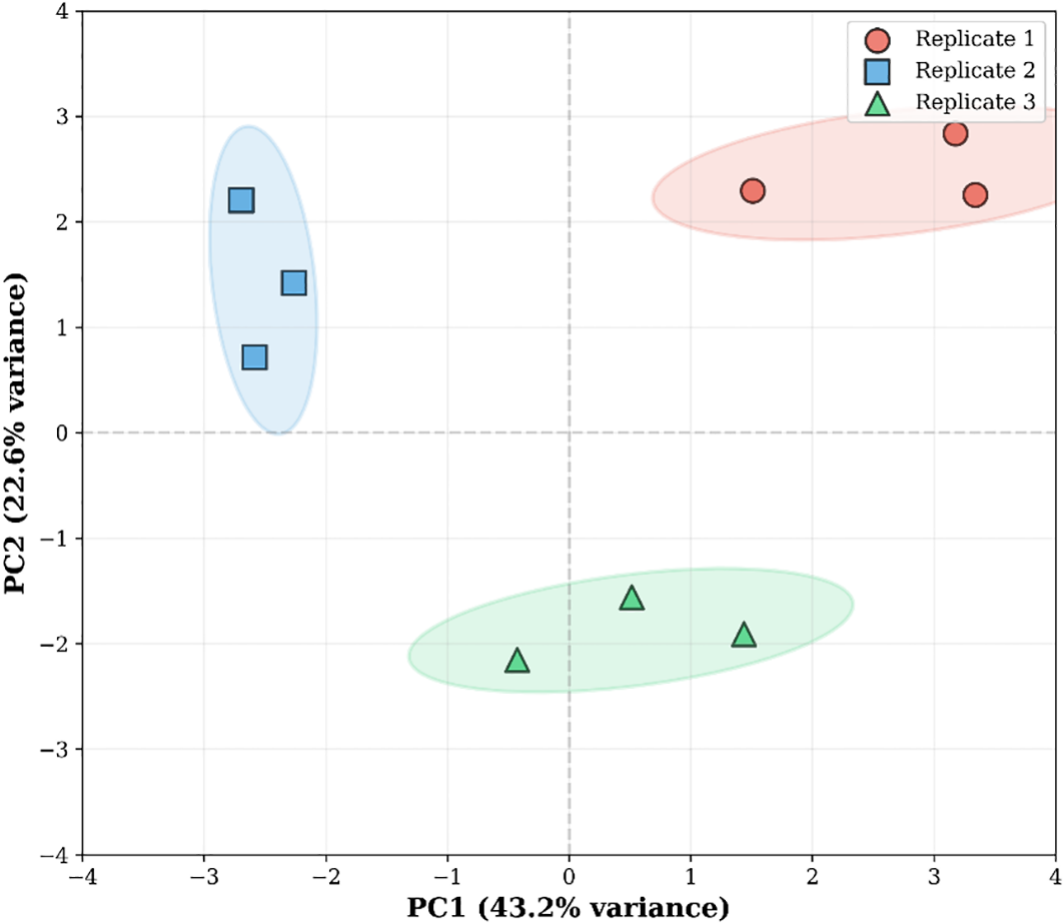
Principal component analysis of lipid related profiles in *S. lasiocarpum* fruit.

To assess the stability of fatty acid composition across different replicates, principal component analysis (PCA) was further conducted on the fatty acid data (**Figure 8**). PC1 and PC2 collectively accounted for approximately two-thirds of the total variance, with PC1 primarily differentiating the overall fatty acid profiles among the three replicates, and PC2 capturing certain secondary variations. The three replicates formed distinct, separated clusters in the PCA space, while sample points within the same replicate clustered tightly, indicating strong consistency between technical and biological replicates and high reliability of the fatty acid quantification results.

#### 3.4.3 Secondary metabolism network

**Figure 9:**
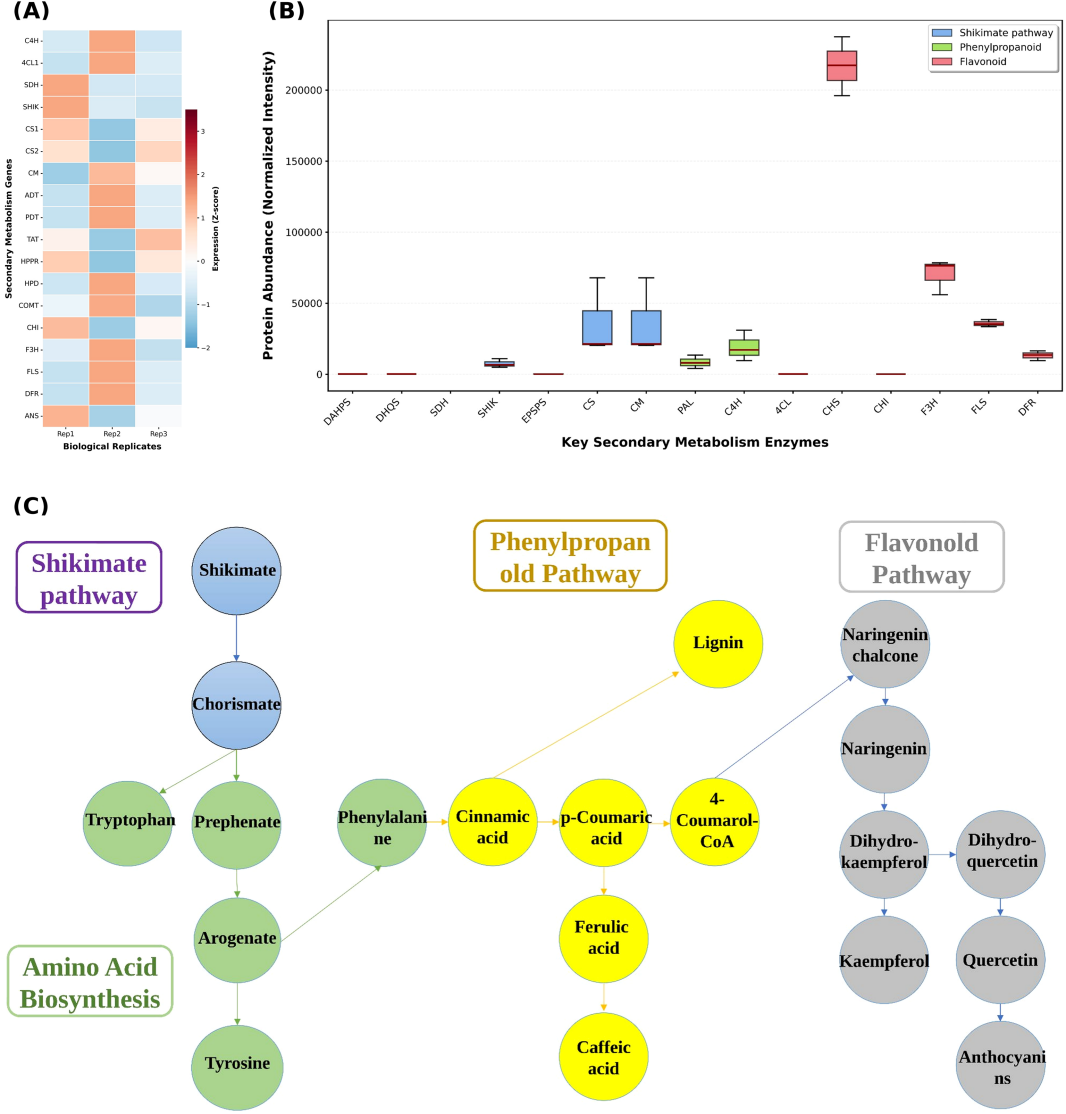
Integrated view of shikimate, phenylpropanoid and flavonoid pathways in *S. lasiocarpum* fruit. **(A)** Heatmap of secondary metabolism genes grouped into shikimate, phenylpropanoid, and flavonoid pathway categories, showing Z score normalized transcript abundance across three biological replicates. **(B)** Protein abundance distributions of representative enzymes from the three pathway groups, presented as boxplots of normalized proteomic intensity. **(C)** Simplified metabolic network linking shikimate pathway intermediates, phenylpropanoid acids, lignin branch metabolites and flavonoid end products, summarizing the connections inferred from transcript, protein, and metabolite annotation.

At the transcriptomic level, aromatic amino acid synthesis and its downstream secondary metabolic pathways were generally maintained at high activity in mature fruit. Genes encoding enzymes in the shikimate pathway, including *DAHPS1*, *DAHPS2*, *DHQS*, *DHD*, *SDH*, *SHIK* and *EPSPS*, showed stable positive Z-scores across the three biological replicates, indicating that the expression of the pathway was overall in a medium-high state. Phenylpropanoid pathway genes derived from aromatic amino acids, including *PAL1*, *PAL2*, *C3H*, *4CL1* and *4CL2*, exhibited continuous high expression bands in the heatmap, suggesting substantial flux from phenylalanine to cinnamic acid and its derivatives. In the flavonoid synthesis pathway, *CHS1*, *CHS2*, and *CHI*, displayed significantly elevated transcript levels. On the other hand, *F3H*, *FLS*, *DFR*, *ANS* and *UFGT* maintained moderately high expression. The patterns among the three biological replicates were highly consistent, indicating that the entire reaction chain related to flavonoid and anthocyanin synthesis was chronically activated in the fruit (**Figure 9A**).

Proteomic data further validated the importance of these key enzymes in the secondary metabolic network. Shikimate pathway enzymes including *DAHPS*, *DHQS*, *SDH*, *SHIK*, and *EPSPS*, showed concentrated abundance distributions across the three biological replicates. The overall boxplot was in a relatively high range, demonstrating that this pathway was also stably expressed at the protein level. Protein abundances of *PAL*, *C4H*, and *4CL*, in the phenylpropanoid pathway were generally higher than most shikimate enzymes, indicating high conversion efficiency from aromatic amino acids to hydroxycinnamic acid intermediates. In the flavonoid synthesis branch, *CHS*, *CHI*, *F3H*, *FLS*, and *DFR* possessed the highest set of abundance values. The distribution ranges between pairs partially overlapped, suggesting that multiple sequential reactions exhibited coordinated elevation at the protein level, ensuring continuous generation of flavonoid scaffolds in the fruit (**Figure 9B**).

Integration of the above information into the metabolic network resulted in a clearer view of the connections between pathways. Shikimate is converted via chorismate to aromatic amino acids. Phenylalanine is then transformed through *PAL*, *C4H*, and *4CL* into a series of hydroxycinnamic acids and their acyl-CoA derivatives. Part of the carbon flux enters lignin synthesis. Another portion is channeled through flavonoid synthase systems to form flavanones, flavanols, and anthocyanin compounds (**Figure 9C**). Transcriptome and proteome data together demonstrated that the upstream shikimate pathway, the phenylpropanoid pathway, and the flavonoid branch, formed a continuous and efficient metabolic route in the mature fruit, indicating that *S. lasiocarpum* fruit continued to invest carbon and nitrogen sources in the synthesis of phenylpropanoid and flavonoid secondary metabolites during its late developmental stage.

## 4 Discussion

### 4.1 Metabolomic findings in phytochemical studies of *S. lasiocarpum*

The metabolite classes detected here broadly agree with earlier reports showing high phenolic and flavonoid content in *S. lasiocarpum*, especially in aqueous extracts optimized for antioxidant recovery (Ab Rahman et al., 2019; Ibrahim et al., 2022). Those extraction-based studies demonstrated strong bioactivity but did not identify the biosynthetic routes involved. Similar chemical patterns have been described in other *Solanum* species, including cultivated eggplant (i.e., *Solanum melongena*), where phenolic acids and flavonoids dominate the fruit metabolome (Plazas et al., 2013; Zhou et al., 2023; Singh et al., 2024). However, the relatively strong representation of shikimate-associated metabolites in *S. lasiocarpum* suggests a pronounced metabolic coupling between primary carbon metabolism and secondary metabolite production. This may help explain both the fruit’s distinctive sour taste and its traditional use primarily as a flavoring ingredient rather than a bulk vegetable (Lim, 2013; Ting and Ding, 2021).

These metabolic differences between *S. lasiocarpum* and *S. melongena* are likely due to the contrasting domestication histories of the two species. While *S. lasiocarpum* shares a close genetic lineage with *S. melongena*, the common eggplant has been extensively bred for fleshy texture and mild flavor. This selection process often inadvertently reduces the accumulation of bitter or astringent secondary metabolites (Gramazio et al., 2014). In contrast, *S. lasiocarpum* retains a phytochemical landscape typical of wild relatives. Our data suggest that this species directs a substantial portion of its carbon flux toward preserving complex defense-related compounds rather than bulk carbohydrate storage. This trade-off accounts for its characteristic sourness and bioactivity, traits that distinguish it from its culinarily tamed relatives (Meyer et al., 2012). Unlike the anthocyanin-dominated profile of purple eggplants, the yellow-fruit phenotype of *S. lasiocarpum* appears to favor upstream hydroxycinnamic acid derivatives, a pattern often observed in wild relatives adapted to higher biotic stress (Chapman and Burke, 2012; Prohens et al., 2013). This preservation of an ancestral metabolic architecture highlights its potential as a reservoir of therapeutic phytochemicals that may have been eroded in major crops (Cornara et al., 2009).

### 4.2 Transcriptional activation of key rate-limiting enzymes in the shikimate pathway combined with expression of central carbon metabolism jointly supports enhanced secondary metabolism

Key enzyme genes positioned at the gateway of the shikimate pathway exhibited active expression. *EPSPS* serves as a crucial junction from shikimic acid to EPSP. Its expression ensured material supply toward aromatic amino acids (Yokoyama et al., 2021). Prephenate dehydratase (*PDT*) in phenylalanine biosynthesis and *PAL* at the start of the phenylpropanoid pathway provided branch amplifiers, effectively channeling carbon flux into the aromatic product series (Yoo et al., 2021). In parallel, expression of central carbon metabolism-related genes (such as *CS*, *IDH*, and *MDH*) and genes related to sugar metabolism/sucrose metabolism (such as *TPS*) furnished substrate and energy/reducing power support for this branch. The hierarchical expression pattern of upstream supply, midstream valve, and downstream amplification forms mutual validation with the appearance of shikimate clusters in the metabolome, suggesting that this pathway is not passively exposed to substrate accumulation but has been subjected to active transcriptional regulation. It should be emphasized that transcriptional activation and flux elevation are not equivalent (Hanson et al., 2021). They are influenced by feedback inhibition (such as negative regulation of upstream synthetic genes by aromatic amino acids), enzyme multimer assembly, and cofactor availability. However, under conditions where multiple gene nodes are coordinately upregulated and energy and precursors are sufficient, channeling effects are expected to improve intermediate transfer efficiency, thereby explaining the metabolite accumulation we observed at the molecular level.

Integrated transcriptome-proteome analysis revealed multi-layered characteristics of metabolic regulation in *S. lasiocarpum* fruit. Plant-specific alternative splicing (especially intron retention) generates a large number of non-functional transcripts to finely regulate protein abundance (Vélez-Bermúdez and Schmidt, 2014). This explains the incomplete consistency between some highly expressed genes (Álvarez-Urdiola et al., 2025) (such as *EPSPS* at 76.5 FPKM and *PAL* at 107.1 FPKM) and their protein detection intensities (Ponnala et al., 2014). During metabolic reprogramming stages, mRNA-protein correlation is significantly weakened, and post-transcriptional regulation becomes the dominant force (Vogel and Marcotte, 2012). Gene co-expression network analysis provides a systems perspective for understanding secondary metabolic regulation. Studies in tea plants and other medicinal plants using WGCNA have shown that transcription factors such as *MYB*, *bHLH*, and *WRKY* (Xie et al., 2024) act as hub genes to coordinately regulate the shikimate pathway (Tai et al., 2018) and flavonoid biosynthesis (Zhu et al., 2024). The coordinated expression patterns of the 2, 159 central carbon metabolism genes and the 8, 248 shikimate-phenylpropanoid-flavonoid pathway genes detected in this study suggest a transcription factor-mediated multi-pathway integrated regulatory mechanism. Comparative genomics studies among *Solanum* species have shown that genes (Messeder et al., 2024) in the shikimate pathway and flavonoid biosynthesis are highly conserved evolutionarily (Xie et al., 2024). Recent work showed that *S. lasiocarpum* fruit extract exhibited significant hypoglycemic activity (α-glucosidase inhibition IC50 = 6.31 mg/mL), which corroborates the high-abundance secondary metabolic enzyme system revealed in this study (Zhao et al., 2025). In addition, related species such as *S. anguivi* and *S. torvum* also showed similar antidiabetic metabolite accumulation characteristics (Nakitto et al., 2021). Metabolic flux analysis indicates that flux regulation in the shikimate pathway is shared by multiple enzymes rather than a single rate-limiting step. Enzymes such as DAHPS, chorismate mutase, and ADT coordinately control the allocation of 30% of photosynthetically fixed carbon toward aromatic amino acids through metabolite feedback inhibition mechanisms (Yoo et al., 2021). This distributed regulation ensures dynamic coordination between primary and secondary metabolism.

### 4.3 Shikimate-derived metabolites may contribute to antidiabetic relevance through a putative *TNF–PPARG* axis

Within the mechanistic framework for treating diabetes, shikimic acid is regarded as a marker of upstream active ingredient sources. Our network pharmacology and PPI prioritisation linked the downstream phenolic acid/flavonoid pool to a putative dual-node axis centred on *TNF* and *PPARG*. On one hand, TNF induces aberrant serine phosphorylation of IRS-1 through the IKK/NF-κB and JNK pathways, weakens the PI3K-*AKT-GLUT4* axis, and amplifies low-grade chronic inflammation in the liver and adipose tissue (Hotamisligil, 2017). This represents an important driver of insulin resistance. On the other hand, PPARG, as a key nuclear receptor for lipid metabolism and insulin sensitivity, can enhance adiponectin expression, promote fatty acid uptake and storage, reduce circulating free fatty acids and inflammation levels (Wang et al., 2014). Clinically, thiazolidinedione drugs achieve hypoglycemic effects through their activation of PPARG. A clear antagonistic relationship exists between the two. Upregulation of *TNF* is often accompanied by suppression of *PPARG* function, while activation of PPARG can negatively regulate *TNF* and its downstream inflammatory factors. Based on metabolomic and upstream transcriptional/proteomic evidence, polyphenols derived from the shikimic acid pathway can inhibit the *TNF*-NF-κB axis through antioxidant and anti-inflammatory effects. They may simultaneously enhance the expression or function of *PPARG* and its target genes, thus forming synergy in the two directions of anti-inflammation and insulin sensitization (Fang et al., 2008). At the tissue level, this dual-target axis is expected to promote differentiation and insulin responsiveness in adipose tissue, reduce lipid deposition and inflammation in the liver, increase GLUT4 translocation and glucose uptake in skeletal muscle, and lower postprandial blood glucose peaks in the intestine through partial phenolic inhibition of glycosidase activity (Kumar et al., 2011). Recent functional food studies illustrate the value of combining phytochemical profiling with mechanistic endpoints when discussing diabetes relevance. For example, red date fruit vinegar has been evaluated together with computational pathway analysis in adults with T2DM and dyslipidaemia (Ali et al., 2025), while targeted metabolomics coupled with gut microbiota readouts has been used to connect phenylpropanoid-rich plant materials with metabolic improvements in diabetic mice (Cheng et al., 2025). These reports provide a useful benchmark for the type of downstream validation that would strengthen the hypotheses generated here. Validation pathways can proceed step by step from cells to *in vivo* settings. In macrophage-adipocyte co-culture systems, inhibition of TNF and downregulation of NF-κB activity can be evaluated, and changes in the *AKT*/GLUT4 axis and *PPARG* target genes can be detected (Suganami et al., 2005). In high-fat diet or STZ models, OGTT/ITT, HOMA-IR, serum inflammatory profiles, and tissue pathology can be observed (Srinivasan et al., 2005). Chemical inhibition or siRNA can be used to blunt *TNF* or *PPARG* separately, testing the dependence and reversibility of the shikimic acid action. At the formulation and translational level, an enriched polyphenol panel and a stable fingerprint are needed to ensure batch-to-batch consistency. Attention must be paid to the impact of oral absorption, first-pass metabolism, and gut microbiota transformation on effective exposure, while also conducting long-term safety monitoring (Ozdal et al., 2016). Thus, these results support a testable working model in which shikimate-derived polyphenols may influence diabetes-relevant inflammation–metabolism crosstalk through *TNF-* and *PPARG*-associated signalling. This model now requires stepwise chemical and functional validation before any causal mechanism can be concluded.Proteomic evidence for post-translational regulation of the shikimate pathway.

### 4.4 Proteomic evidence for post-translational regulation of the shikimate pathway

The depth of proteome coverage obtained here highlights the importance of post-translational regulation in shaping flux through the shikimate–phenylpropanoid–flavonoid network. Transcript abundance alone often poorly predicts metabolic output, with previous studies showing that mRNA levels account for less than half of the variation in protein abundance (Li et al., 2017). This shortcoming represents the central role of translation efficiency, protein stability, and post-translational modification in controlling pathway activity. Differences in protein abundance variability among enzymes further suggest that regulatory control is unevenly distributed across the pathway. Enzymes positioned at key metabolic nodes appear to be more tightly or dynamically regulated, consistent with selective flux control rather than uniform pathway regulation. Such patterns are characteristic of complex metabolic networks, where control is concentrated at steps that coordinate carbon allocation. Post-translational mechanisms provide a rapid and flexible means of regulating secondary metabolism. Phosphorylation and ubiquitination are well established as modulators of enzyme activity and turnover in phenylpropanoid and flavonoid biosynthesis (Friso and van Wijk, 2015). For example, phosphorylation of phenylalanine ammonia-lyase reduces catalytic capacity and promotes degradation, enabling swift downregulation of phenylpropanoid flux (Allwood et al., 1999). Chalcone synthase is similarly regulated via ubiquitin-mediated pathways in response to environmental cues, allowing fine control of flavonoid production (Zhang et al., 2017). Taken together, these observations support a layered regulatory model in which transcription defines biosynthetic potential, while post-translational control determines pathway output. Such regulation allows dynamic balancing of carbon flux between primary metabolism and secondary compound synthesis during fruit development and environmental adjustment.

## 5 Conclusion

In this study, multi-omics analysis revealed that the mature fruit of *S. lasiocarpum* functions as a specialised biosynthetic system that favours the accumulation of shikimate-derived metabolites alongside active primary carbon metabolism. Integration of metabolomic, proteomic, and transcriptomic data revealed a coherent molecular framework supporting sustained phenolic and flavonoid biosynthesis at mature stages. Network pharmacology further linked a subset of annotated compounds to diabetes-relevant human targets and highlighted a putative *TNF–PPARG*-associated axis, which may help to rationalise the fruit’s traditional use in diabetes management. Future work should validate key metabolites using authentic standards and evaluate bioactivity in appropriate cellular and *in vivo* models, followed by well-designed, long-term human intervention studies to clarify efficacy and mechanism in humans.

## Data Availability

The datasets presented in this study can be found in online repositories. The names of the repository/repositories and accession number(s) can be found below: https://www.ncbi.nlm.nih.gov/, PRJNA1304227 https://massive.ucsd.edu/ProteoSAFe/static/massive.jsp, MSV000098900.
