# Supplementary material for: Integrated metabolomic, proteomic, and transcriptomic analyses reveal the production of bioactive metabolites and antidiabetic effects in mature Solanum lasiocarpum fruit
Source: Front Plant Sci. 2026 Mar 6;17:1774981. doi: 10.3389/fpls.2026.1774981 (PMC13002610; doi:10.3389/fpls.2026.1774981)
Supplement: Supplementary file 1 [file Table1.docx]

Table S1 Final active components

| **No.** | **MZ** | **Compounds** | **Adduct** | **Formula** | **Super Class** |
| --- | --- | --- | --- | --- | --- |
| 1 | 584.28 | Dihydroegotamine | (M^+^H)^+^ | C_33_H_37_N_5_O_5_ | Alkaloids and derivatives |
| 2 | 93.03 | Phenol | (M^-^H)^-^ | C_6_H_6_O | Benzenoids |
| 3 | 205.16 | 4-(1,1,3,3-Tetramethylbutyl)-phenol | (M^-^H)^-^ | C_14_H_22_O | Benzenoids |
| 4 | 421.15 | Losartan | (M^-^H)^-^ | C_22_H_23_ClN_6_O | Benzenoids |
| 5 | 230.02 | BETAMIPRON | (M^+^K^-^2H)^-^ | C_10_H_11_NO_3_ | Benzenoids |
| 6 | 314.14 | feruloyltyramine | (M^+^H)^+^ | C_18_H_19_NO_4_ | Benzenoids |
| 7 | 307.10 | Fluconazole | (M^+^H)^+^ | C_13_H_12_F_2_N_6_O | Benzenoids |
| 8 | 186.09 | 1-Naphthaleneacetamide | (M^+^H)^+^ | C_12_H_11_NO | Benzenoids |
| 9 | 297.15 | aurapten | (M^-^H)^-^ | C_19_H_22_O_3_ | Lipids and lipid-like molecules |
| 10 | 277.22 | alpha-Linolenic acid | (M^-^H)^-^ | C_18_H_30_O_2_ | Lipids and lipid-like molecules |
| 11 | 480.31 | LysoPE(18:0/0:0) | (M^-^H)^-^ | C_23_H_48_NO_7_P | Lipids and lipid-like molecules |
| 12 | 474.26 | LysoPE(0:0/18:3(9Z,12Z,15Z)) | (M^-^H)^-^ | C_23_H_42_NO_7_P | Lipids and lipid-like molecules |
| 13 | 496.35 | LysoPC(16:0/0:0) | (M^+^H)^+^ | C_24_H_50_NO_7_P | Lipids and lipid-like molecules |
| 14 | 509.46 | Palmitoleic acid | (2M^+^H)^+^ | C_16_H_30_O_2_ | Lipids and lipid-like molecules |
| 15 | 256.27 | Palmitic amide | (M^+^H)^+^ | C_16_H_33_NO | Lipids and lipid-like molecules |
| 16 | 313.28 | MG(0:0/16:0/0:0) | (M^+^H^-^H_2_O)^+^ | C_19_H_38_O_4_ | Lipids and lipid-like molecules |
| 17 | 415.29 | Deoxycholic acid | (M^+^Na)^+^ | C_24_H_40_O_4_ | Lipids and lipid-like molecules |
| 18 | 282.08 | Guanosine | (M^-^H)^-^ | C_10_H_13_N_5_O_5_ | Nucleosides, nucleotides, and analogues |
| 19 | 235.08 | Didanosine | (M^-^H)^-^ | C_10_H_12_N_4_O_3_ | Nucleosides, nucleotides, and analogues |
| 20 | 87.01 | Pyruvate | (M^-^H)^-^ | C_3_H_4_O_3_ | Organic acids and derivatives |
| 21 | 114.05 | D-Proline | (M^-^H)^-^ | C_5_H_9_NO_2_ | Organic acids and derivatives |
| 22 | 111.04 | (R)-3-methyl-2-oxo-Pentanoic acid | (M^-^H_2_O^-^H)^-^ | C_6_H_10_O_3_ | Organic acids and derivatives |
| 23 | 141.01 | Oxoadipic acid | (M^-^H_2_O^-^H)^-^ | C_6_H_8_O_5_ | Organic acids and derivatives |
| 24 | 111.01 | Glutaconic acid | (M^-^H_2_O^-^H)^-^ | C_5_H_6_O_4_ | Organic acids and derivatives |
| 25 | 164.07 | L-Phenylalanine | (M^-^H)^-^ | C_9_H_11_NO_2_ | Organic acids and derivatives |
| 26 | 189.04 | 3-Dehydroquinic acid | (M^-^H)^-^ | C_7_H_10_O_6_ | Organic acids and derivatives |
| 27 | 116.07 | L-Proline | (M^+^H)^+^ | C_5_H_9_NO_2_ | Organic acids and derivatives |
| 28 | 175.12 | L-Arginine | (M^+^H)^+^ | C_6_H_14_N_4_O_2_ | Organic acids and derivatives |
| 29 | 166.09 | D-Phenylalanine | (M^+^H)^+^ | C_9_H_11_NO_2_ | Organic acids and derivatives |
| 30 | 107.09 | Isobutyric acid | (M^+^NH_4_)^+^ | C_4_H_8_O_2_ | Organic acids and derivatives |
| 31 | 93.07 | Propionic acid | (M^+^NH_4_)^+^ | C_3_H_6_O_2_ | Organic acids and derivatives |
| 32 | 425.18 | Clindamycin | (M^+^H)^+^ | C_18_H_33_ClN_2_O_5_S | Organic acids and derivatives |
| 33 | 120.08 | 2-Hydroxyphenethylamine | (M^+^H^-^H_2_O)^+^ | C_8_H_11_NO | Organic nitrogen compounds |
| 34 | 318.30 | Phytosphingosine | (M^+^H)^+^ | C_18_H_39_NO_3_ | Organic nitrogen compounds |
| 35 | 173.04 | Shikimic acid | (M^-^H)^-^ | C_7_H_10_O_5_ | Organic oxygen compounds |
| 36 | 191.05 | Quinic acid | (M^-^H)^-^ | C_7_H_12_O_6_ | Organic oxygen compounds |
| 37 | 218.10 | Pantothenic acid | (M^-^H)^-^ | C_9_H_17_NO_5_ | Organic oxygen compounds |
| 38 | 149.04 | Glyceraldehyde | (M^+^CH_3_COO)^-^ | C_3_H_6_O_3_ | Organic oxygen compounds |
| 39 | 87.05 | Erythritol | (M^+^H^-^2H_2_O)^+^ | C_4_H_10_O_4_ | Organic oxygen compounds |
| 40 | 153.03 | Hydantoin-5-propionic acid | (M^-^H_2_O^-^H)^-^ | C_6_H_8_N_2_O_4_ | Organoheterocyclic compounds |
| 41 | 177.04 | 3,7-Dimethyluric acid | (M^-^H_2_O^-^H)^-^ | C_7_H_8_N_4_O_3_ | Organoheterocyclic compounds |
| 42 | 245.09 | butalbital | (M^+^Na^-^2H)^-^ | C_11_H_16_N_2_O_3_ | Organoheterocyclic compounds |
| 43 | 144.08 | Tryptophanol | (M^+^H^-^H_2_O)^+^ | C_10_H_11_NO | Organoheterocyclic compounds |
| 44 | 314.09 | Flunitrazepam | (M^+^H)^+^ | C_16_H_12_FN_3_O_3_ | Organoheterocyclic compounds |
| 45 | 163.04 | trans-Caffeic acid | (M^+^H^-^H_2_O)^+^ | C_9_H_8_O_4_ | Phenylpropanoids and polyketides |
